# Interventions for Nursing Home Residents with Dysphagia—A Scoping Review

**DOI:** 10.3390/geriatrics6020055

**Published:** 2021-05-21

**Authors:** Dorte Melgaard, Albert Westergren, Conni Skrubbeltrang, David Smithard

**Affiliations:** 1 Department of Clinical Medicine and Centre for Clinical Research, Aalborg University and North Denmark Regional Hospital, 9800 Hjoerring, Denmark; 2 The Research Platform for Collaboration for Health and the PRO-CARE Group, Faculty of Health Science, Kristianstad University, 291 88 Kristianstad, Sweden; albert.westergren@hkr.se; 3 Department of Health Sciences, Faculty of Medicine, Lund University, 221 00 Lund, Sweden; 4 Medical Library, Aalborg University Hospital, 9000 Aalborg, Denmark; cs@rn.dk; 5 Queen Elizabeth Hospital, Woolwich, London SE18 4QG, UK; david.smithard@nhs.net; 6 Department of Sports Science, University of Greenwich, London SE5 9RS, UK

**Keywords:** care facility, dysphagia, nursing home, nutrition, swallowing disorders, pneumonia

## Abstract

Oropharyngeal dysphagia is common in nursing home residents. The objective of this scoping review was to summarize and disseminate the findings from the literature on interventions for dysphagia in nursing home residents. Searches were conducted in four databases. The criteria for including the studies were nursing home residents, dysphagia, interventions, original research, published in English, Danish, Norwegian, or Swedish with no restriction placed regarding publication date. Excluded were literature reviews, editorial comments, conference abstracts, protocols, papers not available in full text, and studies with a mixed population, for example, geriatric patients and nursing home residents and where the results were not separated between the groups. A total of 14 papers were included and analyzed. The included papers represented interventions focusing on feeding intervention, oral hygiene, caregiver algorithm, stimulation (taste and smell), teaching the residents what to eat, mobilization of the spine, exercises/training, and positioning. This scoping review identifies sparse knowledge about interventions affecting nursing home residents’ dysphagia. But the results indicate that multi-component interventions, including staff training, training of residents, and/or next of kin, might be successful. This scoping review clarifies that there is a need for well-designed studies that uncover which specific interventions have an effect in relation to nursing home residents with dysphagia and can serve as a guide for designing multi-component person-centered intervention studies. Future studies should implement high evidence study designs, define the measures of dysphagia, and quantify the severity of dysphagia, its underlying diseases, and comorbidities.

## 1. Introduction

Oropharyngeal dysphagia (OD) is common in older, particularly frail, people [1]. Multiple age-related changes, including loss of muscle mass and weakness (sarcopenia), changes of the cervical spine, xerostomia triggered due to medication or illness, impaired dental status, and reduced oral and pharyngeal sensitivity, increase the risk of OD [2,3]. OD fulfils the definition of a geriatric syndrome, with a high prevalence in older people, a combination of symptoms, common risk factors (functional or cognitive impairments), interactions with other geriatric syndromes, and impaired outcomes [1]. It is documented that the prevalence of dysphagia is up to 70% among nursing home residents, and the prevalence is highest in patients with severe dementia and neurological diseases [4,5,6,7,8,9]. A wide range of parameters affects the swallowing function in the very elderly, for example, frailty, impaired dental status, polypharmacy, reduction of saliva production, age, and impaired cognition [2,6,10,11,12]. The consequences of OD, generally and particularly in nursing home residents, include malnutrition, dehydration, pneumonia, frailty, and social isolation [1,13,14]. Pneumonia is the second-most common infection occurring in nursing home residents [15]. Pneumonia is common in the presence of dysphagia but is not directly associated with aspiration alone; a sudden inhalation of a large bolus results in choking and, on occasion, death in the nursing home residents [16,17,18,19]. Studies document high mortality among nursing home residents with OD [6,20,21,22]. Two independent studies found that mortality for nursing home residents was greater among those with rather than those without OD, 24.7% vs. 11.9% (*p* < 0.001). Another study documented a mortality rate of 27.7% vs. 16.8% (*p* = 0.0001) [20,22].

Older people are often not aware of their swallowing dysfunctions, and despite the high risk of life-threatening complications, OD is often not detected and treated [2]. Treatment of OD has the main goal of reducing aspiration pneumonia, undernutrition, dehydration, morbidity, decreased quality of life, and mortality. Few studies document the effect of managing OD generally [23,24]. The scientific literature offers limited evidence concerning the best management of OD in nursing home residents. This scoping review aims to map current literature to identify potential gaps in research, both with respect to methodology and in the management of OD in nursing home residents.

## 2. Materials and Methods

Scoping reviews provide descriptive documentation of the existing knowledge of a subject and provides the ability to synthesize and examine the extent and nature of the research [25]. In contrast to a systematic review, the scoping review does not undertake a critical appraisal of individual studies [26]. The present scoping review was conducted using Levac et al.’s methodological framework [25]. The six-stage framework includes identifying the research question, identifying relevant studies, study selection, charting the data, collating, summarizing, and reporting results and consultation [25].

According to stage 1, the research question was ‘what current literature exists according to the management of OD in nursing home residents and is it possible to identify potential gaps in the research?’. The question was kept broad to capture a large amount of information about the topic, but at the same time specific to keep the focus on the challenges there are in treating nursing home residents [25]. In stage 2, the relevant studies were identified, and this process was guided by the research question and performed by an experienced librarian in close collaboration with the authors experienced in the field [25]. In stage 3, the selected studies were screened. This part of the process was a team process, where two authors independently reviewed the title and abstract and afterwards the full paper. If there were any conflicts, these were addressed and solved by the authors [25]. Charting the data in stage 4 was an interactive process involving the authors, and the reported themes that emerged, and a content analysis was completed [25]. In stage 5, an analytic framework was made to provide an overview of the literature. The themes were coded during the process, and the results were reported [25]. The final sixth step in Levac’s framework is optional and includes the possibility of involving consumer and stakeholders; this was considered irrelevant in this context.

### 2.1. Literature Search

A systematic literature search was conducted using PubMed, Embase.com, Cochrane Library, and CINAHL (Ebsco with full text). The search strategy was developed by an experienced medical librarian and reviewed by the research group. The search strategy was developed using block building technique with Boolean operators. Where possible and relevant, proximity operators were also used. The strategy consisted of two blocks, one for Dysphagia and its synonyms truncated and combined with OR using both subject headings and textword: ((‘dysphagia’/exp OR dysphagia* OR presbyhpagia* OR (deglutition OR swallowing) NEAR/3 disorder* OR dysfunction* OR problem* OR difficult*)); and one for nursing home and its synonyms: (‘nursing home’/exp OR “nursing home*” OR “convalescen* home*” OR “care facility*” OR residental*). Finally, the two blocks were combined with AND.

Keywords and subject headings were found by checking the indexing of known relevant articles, the thesauruses’ entry terms, and suggestions from the researchers.

The search strategy was tested and finalized in Embase.com and then translated into the other databases using their equivalent subject headings, all text words, and with syntax adapted accordingly.

**Figure 1:**
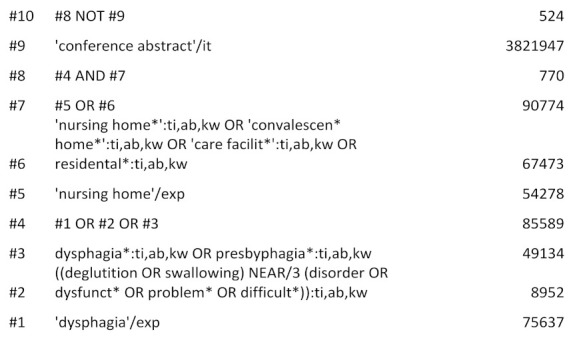
Search strategy Embase.

The databases were searched from their inception to 19 August 2020. The results were exported to and de-duplicated in Endnote X9 bibliographic software. The search strategy for Embase.com is shown in Figure 1 below. The full search strategies for each data-base are detailed in the Appendix A.

### 2.2. Inclusion and Exclusion Criteria

Articles meeting all the following criteria were included in this scoping review: nursing home residents; dysphagia; interventions; original research; published in English, Danish, Norwegian, or Swedish. No restriction was placed regarding publication date. Exclusion criteria were literature reviews, editorial comments, conference abstracts, protocols, papers not available in full text, and studies with a mixed population; for example, geriatric patients and nursing home residents, and where the results were not separated between the groups.

### 2.3. Knowledge Synthesis

The following data was extracted: author(s) and year of publication, country, aims of the study, study design, inclusion and exclusion criteria, intervention, data collection and analysis methods, study participants, primary outcomes and results.

## 3. Results

**Figure 2:**
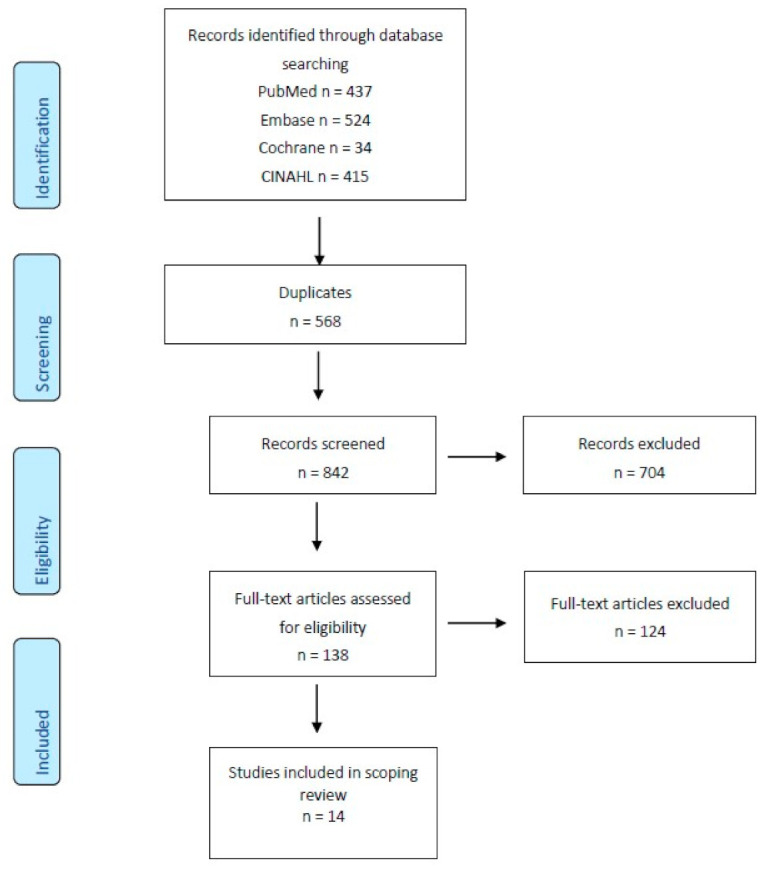
PRISMA flow diagram for scoping reviews, adapted from Tricco et al. [27]. The included studies took place in the United States (*n* = 6), Taiwan (*n* = 2), Canada (*n* = 1), the Netherlands (*n* = 1), Japan (*n* = 1), Korea (*n* = 1), Italy (*n* = 1), and Belgium (*n* = 1).

The initial search resulted in 973 articles for review as presented in the PRISMA flow diagram (Figure 2, Table S1). After removal of duplicates, and screening of titles and abstracts, the review was conducted for 138 full-text articles, and 14 articles met inclusion and exclusion criteria and were included for review.

The 14 included studies are presented and summarized in Table 1.

The studies could be categorized according to the type of interventions. Due to the heterogeneity of the included studies reviewed, the intervention categories are broad with some overlap. Three studies described a feeding intervention [32,38,41], two studies focused on oral hygiene [40,42], two studies on stimulation (taste and smell) [30,31], two studies on training [28,29], two studies on a caregiver algorithm [35,36], [43], one study focused on teaching the residents compensatory strategies [37], one study focused on mobilization of the spine [33], and one study focused on positioning, oral hygiene, and teaching swallowing techniques [34].

### 3.1. Feeding Intervention

The effect of a feeding intervention in nursing home residents was evaluated in three studies [32,38,41]. Studies testing the effect of modified textures, optimizing the preparation of the resident and the surroundings documented significantly improved food intake [38,41]. A study offering five instead of three meals documented a similar average energy intake between the three and five meal patterns [32].

### 3.2. Oral Hygiene

In two studies, the focus was on oral hygiene [39,40]. A controlled clinical trial evaluated the effect of adding a 0.05% chlorhexidine-containing solution to daily oral hygiene care, but no significant difference in the incidence of pneumonia was documented [39]. A feasibility study including four nursing home residents tested a mouth care protocol, including mouth care twice daily and once on day 6, which resulted in improved oral hygiene without aspiration [40]. Thus, the role of nurses in providing oral care seems of importance for resident safety, i.e., for protection against aspiration.

### 3.3. Stimulation—Taste and Smell

A controlled clinical trial investigating the effect of sour and sweet-sour taste (citric acid) documented significantly improved swallowing and less aspiration and penetration compared to when the residents drank water. The study also documented that teaspoon delivery of liquids significantly reduced aspiration and penetration compared with natural cup drinking [30]. A randomized, controlled study evaluating the effect of olfactory stimulation with volatile black pepper oil documented significantly shortened latency of the swallowing reflex, compared with lavender oil and distilled water [31].

### 3.4. Training

An intervention study determined the effects of swallowing therapy where the residents (all with PEG tube) were doing swallowing therapy (e.g., tongue-resistance and retraction exercises, range of motion exercises of lips, jaw, and tongue) or compensatory techniques (e.g., chin-down posture) 20 min twice daily in a period ranging from 2–16 weeks. After the therapy, oral feeding was introduced in all patients and PEG tubes were removed in 10 of the 16 patients, and weight and albumin levels increased [28]. A quasi-experimental parallel cluster-designed study examined the effect of training on functional swallowing. A significant effect was reached for residents receiving a structured swallowing training program 30 min each day for 6 days per week for 8 weeks according to volume per second, volume per swallow, mid-arm circumference, and body weight compared to the control group that received no training [29].

### 3.5. Caregiver Algorithm

A randomized controlled study evaluated the effect of a decision aid compared to usual care. Next of kin in intervention sites received a structured decision aid providing information about dementia, feeding options and the outcomes, advantages, and disadvantages of feeding tubes or assisted oral feeding. Next of kins in the control group received usual care, including any information from health care providers. After three months, the next of kins who received the decision aid had significantly lower scores on the Decisional Conflict Scale than next of kins receiving usual care [35].

A control-intervention study examined the effect of an evidence-based nursing care algorithm of dysphagia consisting of (1) screening for dysphagia, (2) grouping by the degree of dysphagia risk, and (3) nursing care for each group (checking for OD signs, positioning, instructing in exercises, oral hygiene, meal assisting, and modified diet). Implementing this algorithm improved the dysphagia-related quality of life and reduced the risk of aspiration among the nursing home residents [36]. Thus, the role of nurses in using evidence-based guidelines is of utmost importance for resident safety, i.e., in avoiding aspiration.

### 3.6. Teaching the Residents Compensatory Strategies

A pilot study including five nursing home residents with dementia and OD determined the effect of using compensatory swallowing strategies and spaced retrieval, paired with an external memory aid. The treatment was provided in 30- to 45-min sessions five times weekly in a quiet room. Compensatory strategies were trained using cards with the responses printed. The use of a visual aid was related to functional improvements in 2–3 compensatory swallowing behaviors for each of the five participants [37].

### 3.7. Mobilization of the Spine

A randomized controlled trial with a crossover design investigated the feasibility of cervical spine mobilization in elderly dementia nursing home residents with dysphagia. A physiotherapist mobilized the head and cervical spine to correct the patient’s posture. Three sessions were performed during a ‘mobilization week’ (every two days), each session lasting approximately 20 min and the swallowing capacity improved significantly [33].

### 3.8. Positioning, Oral Hygiene, and Swallowing Techniques

A randomized feasibility study identified the feasibility of a multi-component intervention protocol where the residents were randomized to 1) upright feeding positioning, 2) teaching swallowing techniques, or 3) manual, oral brushing plus 0.12% chlorhexidine. Episodes of cough during swallowing were reduced at the end of three months in six of eight (75%) participants assigned to manual brushing, three of seven (43%) of participants assigned to feeding positioning, and three of seven (43%) of participants assigned to instruction in swallowing techniques. Manual brushing was not significantly more effective than the other two intervention protocols [34].

## 4. Discussion

The aim of this scoping review was to map current literature to identify potential gaps in research in the management of OD in nursing home residents. A total of 15 studies met the review inclusion criteria and described a range of interventions for dysphagia. The heterogeneity across all examined studies was high. Study designs were largely prospective and observational, and only four randomized controlled trials with limited sample sizes ranging from 15 to 256 participants were included [31,33,34,35]. In general, the sample size in the studies was low; 66% of the studies included fewer than 40 participants.

Different terms for OD are used in the studies, and in many of the studies, it is unclear how OD is defined and whether and how it is assessed. It is often unclear whether dysphagia is defined as oropharyngeal and/or esophageal dysphagia or the broader definition of swallowing disorders. Only one study used a gold standard for assessing dysphagia [30]. The severity of dysphagia or details about comorbidity is in general not well described in the studies as well.

Some studies identified in this review did not provide sufficient information about the intervention and outcome [31,32,33,34,35,40,41,42,43]. Additionally, it was often unclear whether the dysphagia outcome per se was evaluated, what methods were used to determine the outcome, and when it was measured. More clearly documented management or treatment regimens using consistent, standardized outcome measures assist in concluding an intervention’s effectiveness. Robust research is needed for the clinicians and managers to decide what interventions and outcome measures to implement for nursing home residents when prioritizing the often under-funded healthcare services.

Based on the evidence identified, this scoping review demonstrates sparse knowledge about which interventions affect nursing home resident’s dysphagia. It is common practice to serve a texture-modified diet to improve food intake and to prevent aspiration in nursing home residents, but a review document limited evidence for the effect of the intervention [44]. Two studies in this scoping review demonstrate increased BMI when a texture-modified diet was served, but the studies did not measure the effect on aspiration [38,41], It seems that multi-component interventions, including staff training [36], training of residents [28,29], and/or next of kin [35], might be successful. Structured dysphagia screening and decision protocols [35,36] need to be part of such intervention together with good oral care [34,40]. However, due to the complex interaction between the elderly person’s condition, that is, the comorbidity that coexists and interacts with dysphagia, it seems necessary to evaluate further multi-component interventions in which education/training should be one part, as the existing studies show a clear tendency towards the positive effect of efforts in this area [28,29,35,36]. Another relevant focus is polypharmacy. It is well described that a side effect of medication can be dysphagia, but there is a lack of studies on the effects of adapting nursing home residents’ medication [45,46,47,48]. A study detecting the effect of multidisciplinary management of nursing home residents with dysphagia is very relevant, as it is described in more studies that a multidisciplinary approach increases the efficiency and quality of the treatment [49,50,51]. Evaluation of multi-component interventions is a difficult task. It may be difficult to identify what exactly in the combination of interventions has the most effect and what combination of interventions is best. There are ethical demands when conducting such studies. It would be unethical not to provide treatment to the control group, making it impossible to have a control group receiving ‘no treatment’. Thus, there is a need for more well-designed intervention studies, including well-described multi-component interventions. This scoping review can serve as a guide for designing such multi-component intervention studies. There is also a need for studies regarding the effect of dysphagia team collaboration and consultations for nursing home residents since we found no such intervention study.

A limitation in the current scoping review is that only English, Danish, Norwegian, or Swedish publications were included, but not, for example, Japanese and Spanish, even though researchers in this field are very active in both these language areas. Studies not measuring direct effect for the residents were excluded to avoid the inclusion of studies outside the scope of this review. The authors acknowledge the risk of missing information that may have been embedded in the excluded studies. A full-text review was conducted on articles where the title or abstract was unclear, and this minimized the risk of missing information as much as possible.

## 5. Conclusions

Based on the evidence identified, this scoping review demonstrates sparse knowledge about which interventions affect nursing home residents’ dysphagia. Many of the included studies have several limitations, including the definition of dysphagia, description of the interventions, small study samples etc.

Modified textures, oral hygiene, mobilization of the spine and an evidence-based nursing care algorithm seem to improve the swallowing capacity, reduce the risk of aspiration, and improve the quality of life in nursing home residents.

Given the large number of nursing home residents who have dysphagia and the major consequences it has, both for the individual and society, there should be an increased focus on research in this area. Future research should strive for stronger evidence, for example, a randomized, controlled study design with larger sample sizes and multi-component interventions. This scoping review can serve as a guide for designing such a multi-component person-centered intervention study.

## Figures and Tables

**Table 1:** Summary of study details and population (sorted after publication year).

	Study Aim(s)	Study Design, Inclusion and Exclusion Criteria, Intervention, Data Collection and Analysis Methods	Study Participants	Primary Outcomes	Results
Klor, B M et al., 1999 [28] USA	Determining the effects of swallowing therapy.	Intervention study. Inclusion criteria: PEG tube, nonprogressive neurogenic dysphagia (Modified Barium Swallow), neurological and medical stability, and language and cognitive skills. Intervention: treatment with either swallowing therapy (e.g., tongue-resistance and retraction exercises to minimize vallecular residue) and compensatory techniques (e.g., chin-down posture). Individual treatment/training sessions were 20 min twice daily in a period ranging from 2–16 weeks.	*n* = 16. All male.	The ability to take oral feeding.	All patients had positive effect of the treatment, and oral feeding was introduced in all patients. PEG tubes were removed in 10 of the 16 patients. A mean weight gain of 5.1 pounds and a mean albumin increase of 0.5 g/dL were reached.
Lin L et al., 2003 [29] Taiwan	Examining the functional swallowing and nutritional outcomes of swallowing training.	Quasi-experimental parallel cluster design. 7 nursing homes. Inclusion criteria: stroke, dysphagia (FEES), oral intake, mentally and linguistically able to participate. Intervention: the intervention group received a structured swallowing training program, 30 min of swallowing training each day for 6 days per week for 8 weeks. The control group: no training.	Control group *n* = 14 and intervention group *n* = 35.	Swallowing volume and swallowing speed.	Mean differences in volume per second, volume per swallow, mid-arm circumference and body weight between pre- and post-training of the experimental group were significantly higher than for the control group, while mean differences in neurological examination and choking frequency during meals for the experimental group were significantly lower than in the control group.
Pelletier C A et al., 2003 [30] USA	Investigate the effect of sour (citric acid) and sweet-sour taste.	Controlled clinical trial. Inclusion criteria: neurologic dysphagia (FEES), thickened liquids, stable medical condition. Intervention: during FEES examination, initially presented with water, sour liquid (2.7% citric acid), and sweet-sour mixture (1.11% citric acid 8% sucrose) in 5 mL and cup drinking.	*n* = 11	Swallowing impairment (PAS scale), stage transition duration, and frequency of spontaneous swallows.	Citric acid improved swallowing (i.e., less aspiration and penetration) compared with water. Teaspoon delivery of liquids significantly reduced aspiration and penetration compared with natural cup drinking.
Ebihara et al., 2006 [31] Japan	Determine the effect of olfactory stimulation with volatile black pepper oil (BPO).	Randomized, controlled study. Inclusion criteria were nursing home residents with physical symptoms and cognitive impairment being stable for the preceding three months, dysphagia (assessment not defined). Exclusion criteria were unstable health conditions. Intervention: (1) BPO-treated group, (2) a lavender oil group, and (3) odorless group.	*n* = 105	Latency of the swallowing reflex (LTSR), the number of swallowing movements, serum substance P, and regional cerebral blood flow.	Nasal inhalation of BPO for 1-min shortened LTSR, compared with that of lavender oil and distilled water (*p* < 0.03). Compared with the period before the study, the 1-month intervention using BPO improved LTSR with an increase of SP (*p* < 0.01). The number of swallowing movements for 1 min during the nasal inhalation of BPO increased (*p* < 0.001).
Taylor, K A et al., 2006 [32] Canada	Assessing energy content in five vs. three daily meals would improve energy intake.	Crossover study. Inclusion criteria: ≥65 years, dysphagia (prior dysphagia symptoms based on bedside evaluation by an experienced swallowing team) and receiving a texture- modified diet. Exclusion criteria: tube-fed, medically unstable, or receiving a diabetic diet. Intervention: the residents were randomly assigned to three or five meals during an initial 4-day study period, followed by the opposite meal pattern in a second period.	*n* = 31	Energy intake.	Average energy intakes were similar between the three- and five-meal patterns (1325 ± 207 kcal/day vs. 1342 ± 177 kcal/day, respectively; *p* = 0.565); fluid intake was higher with five meals (698±156 mL/day) vs. three (612 ± 176 mL/day;*p* = 0.003).
Bautmans, I et al., 2008 [33] Belgium	Investigating the feasibility of cervical spine mobilization in elderly dementia nursing home residents with dysphagia	A randomized controlled trial with crossover design. Inclusion criteria: ≥65 years, Alzheimer’s dementia (MMSE < 24/30), cervical anterior position, extension or kyphosis and dysphagia (speech therapy report in the medical record). Exclusion criteria: central nervous conditions that could influence swallowing, acute illness or fed by a tube. Intervention: a physiotherapist mobilized the head and cervical spine to correct the patient’s posture. Three sessions were performed during the mobilization week (every 2 days), each session lasting approximately 20 min. Control sessions were identical in planning and duration but consisted of a socializing visit by the physical therapist.	*n* = 15	Feasibility (attendance, hostility to therapy, complications) and dysphagia limit (maximal volume of water (0–20 mL) that can be swallowed in a single movement)	90% of cervical spine mobilization sessions were completed successfully, and no complications were observed. Swallowing capacity improved significantly from 3 mL to 5 mL after one session (*p* = 0.01) and to 10 mL after the one-week treatment (*p* = 0.03).
Quaglia- rello, V et al., 2009 [34] USA	Identify the feasibility of a multi-component intervention protocol consisting of three different interventions.	Randomized feasibility study. Inclusion criteria: nursing home residents, >age 65 years, swallowing difficulties (cough during swallowing). Exclusion criteria were residents <4 weeks; residents for short-term rehabilitation only; estimated to survive <6 months, tube-fed or had a tracheostomy. Intervention: participants were assigned to three groups: (1) upright feeding positioning, (2) teaching swallowing techniques, or (3) manual oral brushing.	*n* = 22	Reduction in the frequency of cough during swallowing— quantified by the number of episodes of coughing while eating in the previous week as follows: never, sometimes (<50% of time), frequently (>50% of time), always.	Episodes of cough during swallowing were reduced at the end of 3 months in 6 of 8 (75%) participants assigned to manual brushing, 3 of 7 (43%) of participants assigned to feeding positioning, and 3 of 7 (43%) of participants assigned to instruction in swallowing techniques. Manual brushing was not significantly more effective than the other two intervention protocols (*p* = 0.31).
Hanson, L C et al., 2011 [35] USA	Testing whether a decision aid, compared to usual care, could improve the quality of decision-making by next-of-kins for nursing home residents with advanced dementia.	Randomised controlled study in 24 nursing homes. Inclusion criteria: nursing home resident, > 65 years, advanced dementia, feeding problems (difficulty swallowing, choking on food or liquid, dehydration, dysphagia, or aspiration). Exclusion criteria: feeding tube, enrolled in hospice, or had weight loss associated with diuresis. Intervention: Next of kins in intervention sites received a structured decision aid providing information about dementia and feeding options. Next of kins in the control group received usual care, including any information from health care providers. Next of kins had in-person interviews with trained research assistants at enrolment and telephone interviews at 1 and 3 months. Structured nursing home chart reviews were completed at enrolment, 1 and 3 months.	*n* = 256	Next of kins decisional conflict at 3 months, knowledge about dementia and feeding options, frequency of communication with health care providers, and use of feeding treatments.	Next of kins in both groups experienced the same level of decisional conflict at the time of study enrollment. After 3 months next of kins who received the decision aid had significantly lower (better) scores on the Decisional Conflict Scale than surrogates receiving usual care (1.65 vs. 1.97, *p* < 0.001), and lower scores on each subscale. Examining within-group change, both groups of next of kins experienced reduced decisional conflict over 3-month follow-up. However, those in the intervention arm had a significantly greater reduction of decisional conflicts (*p* < 0.001).
Park et al., 2015 [36] Korea	To examine the effect of an evidence-based nursing care algorithm	Control intervention study.Inclusion criteria were ≥65 years, living on the same floor, remained min. 6 months in the nursing home and oral intake, dysphagia (Gugging Swallowing Screen). A control period of 6 months was followed by an algorithm period of 6 months. Intervention: nursing care algorithm consisting of (1) screening for dysphagia, (2) grouping by the degree of dysphagia risk, and (3) nursing care for each group (checking for OD signs, positioning, instructing in exercises, oral hygiene, meal assisting and modified diet)	*n* = 40	Dysphagia-related quality of life and risk of aspiration	Improvement of dysphagia-related quality of life and reducing the risk of aspiration
Benigas, J et al., 2016 [37] USA	Determine the effect of teaching persons with dementia to use compensatory swallowing strategies paired with an external memory aid.	Pilot study. Inclusion criteria: dementia and dysphagia (FEES). 65 to 99 years, sixth grade or higher level of education, ability to sit at 90° during the sessions, agreement to eat in a quiet environment when working with the investigator, be a verbal communicator. Exclusion criteria: diagnosis of psychiatric illness. Intervention: 30- to 45-min treatment sessions five times weekly in a quiet room, and compensatory strategies were trained using a yellow card with the responses printed.	*n* = 5	The response was considered correct only if all the responses were performed in the correct order.	The use of a visual aid was functionally related to improvements in 2–3 compensatory swallowing behaviors for each of the 5 participants.
Chen L L et al., 2016 [38] Taiwan	Investigate the effects of an optimal feeding intervention in nursing home residents with Alzheimer’s disease and dysphagia.	Prospective cohort study. Inclusion criteria >60 years, dementia, gagging, dysphagia (Kubota water swallow test), able to take food. Exclusion criteria: severe liver, kidney, or blood diseases, other diseases that may have affected swallowing function, dependent on gastric tube or feeding tube for nutrition or unstable vital signs. Intervention: The residents were prepared for the meal; surroundings were optimized in a three-month period. Observations and assessments of the patients were performed pre- and post-intervention	*n* = 30	Swallowing function measured with the Kubota water swallow test, type and amount of food intake, and nutritional status.	Patients’ eating/feeding abilities improved overall, including significantly increased food intake (*p* < 0.001), improvement in the Kubota water swallow test (*p* < 0.001) and significant improvements in skinfold thickness, arm circumference, serum albumin and hemoglobin (all *p* < 0.01), indicating improved nutritional status. Among 22 patients who initially required assisted feeding, 5 patients resumed self-feeding after the intervention (*p* = 0.06).
Hollaar, V et al., 2017 [39] Netherlands	Assessing the effect of a 0.05% chlorhexidine-containing solution in addition to daily oral hygiene care.	Controlled clinical trial. 17 nursing homes. Inclusion criteria: ≥65 years, physically disabled, dysphagia (assessed by a speech therapist). Exclusion criteria: cognitively impaired, vegetative state, terminally ill, dependent on mechanical ventilation or using an additional oral hygiene care solution. Intervention: for 1 year, participants in the intervention group received the usual oral hygiene care with the addition of a 0.05% chlorhexidine oral rinse solution, and participants in the control group received only oral hygiene care.	*n* = 103	Episodes of pneumonia	Survival analysis showed no significant difference in the incidence of pneumonia between both groups. After adjustment for group and FOIS-level, regression analysis showed that the variables age, gender, CDS score, number of diseases, medication use, number of teeth, and the presence of dental implants or removable dentures were not significantly associated with the incidence of pneumonia.
Jablonski R A et al., 2017 [40] USA	Determine the safety of a mouth care protocol for nursing home care residents with dysphagia and no access to suction equipment.	Feasibility study. Inclusion criteria were nursing home residents, ≥65 years, dysphagia (diagnosis of dysphagia in medical record), dementia, dependent on others for mouth care and having min. two teeth. The oral health assessment tool, the Katz index of independence of activity in daily living, and the global deterioration scale was used to collect information. Intervention: mouth care was provided twice daily for 5 days and once for day 6.	*n* = 4	Microbe abundance	The mouth care protocol resulted in improved oral hygiene without aspiration.
Zanini, M et al., 2017 [41] Italy	Assess changes in daily food intake following the introduction of meals with modified textures and an adequate content of proteins, calories, balanced nutritional and bromatological properties.	Intervention study in 20 nursing homes. Inclusion criteria: nursing home residents ≥ 65 years, CIRS-score < 6 and diagnosed with dysphagia (diagnosis of dysphagia in medical record). Exclusion criteria: clinical instability, terminally ill, chronic or cancer diseases, severe dysphagia, DOSS ≥ 2 or tube feeding. Intervention: meals without nutritional supplementation, but with personalised levels of density, viscosity, texture.	*n* = 479	Nutritional status (anthropometric parameters, plasmatic biochemical nutritional parameters, and nutritional screening tools)	The total mean BMI of the sample increased from 17.88 to 19.00; body weight averagely improved by 7.19%. There was a progressive improvement of total protein and serum albumin values. No side effects were reported.

BMI, Body Mass Index; BP, Black Pepper Oil; CDS, Care-Dependency Scale; CIRS, Cumulative Illness Scale; DOSS, Dysphagia Outcome Severity Scale; FEES, Fiber-Optic Endoscopic Evaluation of Swallowing; FOIS, Functional Oral Intake Scale; Katz Index, The Katz Index of Independence of Activities of Daily living; LTSR, Latency of The Swallowing Reflex; MDS, Minimum Data Set; MNA, Mini Nutritional Assessment; PAS, Penetration–Aspiration Scale; PEG, Percutaneous Endoscopic Gastrostomy; SP, Serum Substance P.

## Data Availability

The data presented in this study are openly available in references.

## Supplementary Materials

The following are available online at https://www.mdpi.com/article/10.3390/geriatrics6020055/s1, Table S1: Preferred Reporting Items for Systematic reviews and Meta-Analyses extension for Scoping Reviews (PRISMA-ScR) Checklist.

## Appendix A

### Search Strategies in PubMed

(“Deglutition Disorders”[MeSH Terms] OR “presbyphagia*”[Text Word] OR “deglutition disorder*”[Text Word] OR “swallowing disorder*”[Text Word] OR “dysphagia*”[Text Word] OR “deglutition dysfunct*”[Text Word] OR “swallowing dysfunction*”[Text Word] OR “swallowing difficult*”[Text Word] OR “deglutition difficult*”[Text Word] OR “deglutition problem*”[Text Word] OR “swallowing problem*”[Text Word]) AND (“Nursing Homes”[MeSH Terms] OR “nursing home*”[Text Word] OR “convalescence home*”[Text Word] OR “convalescent home*”[Text Word] OR “care facilit*”[Text Word] OR residental*[Text Word]) Sort by: Publication Date

Result 437

**Table A1:** Embase.com.

No.	Query	Results
#10	#8 NOT #9	524
#9	‘conference abstract’/it	3,821,947
#8	#4 AND #7	770
#7	#5 OR #6	90,774
#6	‘nursing home*’:ti,ab,kw OR ‘convalescen* home*’:ti,ab,kw OR ‘care facilit*’:ti,ab,kw OR residental*:ti,ab,kw	67,473
#5	‘nursing home’/exp	54,278
#4	#1 OR #2 OR #3	85,589
#3	dysphagia*:ti,ab,kw OR presbyphagia*:ti,ab,kw	49,134
#2	((deglutition OR swallowing) NEAR/3 (disorder OR dysfunct* OR problem* OR difficult*)):ti,ab,kw	8952
#1	‘dysphagia’/exp	75,637

**Table A2:** Cochrane Library.

ID	Search	Hits
#1	MeSH descriptor: [Nursing Homes] explode all trees	1334
#2	(“nursing home*” OR “convalescence home*” OR “convalescent home*” OR “care facilit*” OR residental*):ti,ab,kw	3363
#3	#1 OR #2	3803
#4	MeSH descriptor: [Deglutition Disorders] explode all trees	2834
#5	(((deglutition OR swallowing) NEAR/3 (disorder OR dysfunct* OR problem* OR difficult*))):ti,ab,kw	804
#6	(dysphagia* OR presbyphagia*):ti,ab,kw	4057
#7	#4 OR #5 OR #6	6590
#8	#3 AND #7	34

**Table A3:** Cinahl.

Search ID#	Search Terms	Results
S8	S4 AND S7	415
S7	S5 OR S6	69,092
S6	“nursing home*” OR “convalescen* home*” OR “care facilit*” OR residental*	66,004
S5	(MH “Nursing Homes+”)	27,595
S4	S1 OR S2 OR S3	13,469
S3	dysphagia* OR presbyphagia*	8683
S2	((deglutition OR swallowing) N3 (disorder OR dysfunct* OR problem* OR difficult*))	9720
S1	(MH “Deglutition Disorders”)	8508

## References

[1] Baijens L.W., Clave P., Cras P., Ekberg O., Forster A., Kolb G.F., Leners J.C., Masiero S., Mateos-Nozal J., Ortega O. (2016). European Society for Swallowing Disorders—European Union Geriatric Medicine Society white paper: Oropharyngeal dysphagia as a geriatric syndrome. Clin. Interv. Aging.

[2] Wirth R., Dziewas R., Beck A.M., Clave P., Hamdy S., Heppner H.J., Langmore S., Leischker A.H., Martino R., Pluschinski P. (2016). Oropharyngeal dysphagia in older persons—From pathophysiology to adequate intervention: A review and summary of an international expert meeting. Clin. Interv. Aging.

[3] Altman K.W. (2012). Oropharyngeal dysphagia pathophysiology, complications and science-based interventions. Nestle Nutr. Inst. Workshop Ser..

[4] Rofes L., Arreola V., Romea M., Palomera E., Almirall J., Cabre M., Serra-Prat M., Clave P. (2010). Pathophysiology of oropharyngeal dysphagia in the frail elderly. Neurogastroenterol. Motil..

[5] Namasivayam A.M., Steele C.M. (2015). Malnutrition and Dysphagia in long-term care: A systematic review. J. Nutr. Gerontol. Geriatr..

[6] Sarabia-Cobo C.M., Pérez V., De Lorena P., Domínguez E., Hermosilla C., Nuñez M.J., Vigueiro M., Rodríguez L. (2016). The incidence and prognostic implications of dysphagia in elderly patients institutionalized: A multicenter study in Spain. Appl. Nurs. Res..

[7] Langmore S.E., Olney R.K., Lomen-Hoerth C., Miller B.L. (2007). Dysphagia in patients with frontotemporal lobar dementia. Arch. Neurol..

[8] Horner J., Alberts M.J., Dawson D.V., Cook G.M. (1994). Swallowing in Alzheimer’s disease. Alzheimer Dis. Assoc. Disord..

[9] Clave P., Rofes L., Carrion S., Ortega O., Cabre M., Serra-Prat M., Arreola V. (2012). Pathophysiology, relevance and natural history of oropharyngeal dysphagia among older people. Nestle Nutr. Inst. Workshop Ser..

[10] Pu D., Murry T., Wong M.C.M., Yiu E.M.L., Chan K.M.K. (2017). Indicators of dysphagia in aged care facilities. J. Speech-Lang. Hear. Res..

[11] Park Y.-H., Han H.-R., Oh B.-M., Lee J., Park J., Yu S.J., Chang H. (2013). Prevalence and associated factors of dysphagia in nursing home residents. Geriatr. Nurs..

[12] Pu D., Yiu E.M.L., Chan K.M.K. (2020). Factors associated with signs of aspiration in older adults: A prospective study. Geriatr. Nurs..

[13] Serra-Prat M., Palomera M., Gomez C., Sar-Shalom D., Saiz A., Montoya J.G., Navajas M., Palomera E., Clave P. (2012). Oropharyngeal dysphagia as a risk factor for malnutrition and lower respiratory tract infection in independently living older persons: A population-based prospective study. Age Ageing.

[14] Ekberg O., Hamdy S., Woisard V., Wuttge-Hannig A., Ortega P. (2002). Social and psychological burden of dysphagia: Its impact on diagnosis and treatment. Dysphagia.

[15] Nicolle L.E., Strausbaugh L.J., Garibaldi R.A. (1996). Infections and antibiotic resistance in nursing homes. Clin. Microbiol. Rev..

[16] Van der Maarel-Wierink C.D., Vanobbergen J.N., Bronkhorst E.M., Schols J.M., de Baat C. (2011). Meta-analysis of dysphagia and aspiration pneumonia in frail elders. J. Dent. Res..

[17] Almirall J., Cabré M., Clavé P. (2012). Complications of oropharyngeal dysphagia: Aspiration pneumonia. Nestle Nutr. Inst. Workshop Ser..

[18] Hollaar V.R.Y., van der Putten G.-J., van der Maarel-Wierink C.D., Bronkhorst E.M., de Swart B.J.M., de Baat C., Creugers N.H.J. (2017). Nursing home-acquired pneumonia, dysphagia and associated diseases in nursing home residents: A retrospective, cross-sectional study. Geriatr. Nurs..

[19] Quagliarello V., Ginter S., Han L., Van Ness P., Allore H., Tinetti M. (2005). Modifiable risk factors for nursing home-acquired pneumonia. Clin. Infect. Dis..

[20] Wirth R., Pourhassan M., Streicher M., Hiesmayr M., Schindler K., Sieber C.C., Volkert D. (2018). The impact of dysphagia on mortality of nursing home residents: Results from the nutritionDay project. J. Am. Med. Dir. Assoc..

[21] Hoshino D., Watanabe Y., Edahiro A., Kugimiya Y., Igarashi K., Motokawa K., Ohara Y., Hirano H., Myers M., Hironaka S. (2020). Association between simple evaluation of eating and swallowing function and mortality among patients with advanced dementia in nursing homes: 1-year prospective cohort study. Arch. Gerontol. Geriatr..

[22] Jukic Peladic N., Orlandoni P., Dell’Aquila G., Carrieri B., Eusebi P., Landi F., Volpato S., Zuliani G., Lattanzio F., Cherubini A. (2019). Dysphagia in nursing home residents: Management and outcomes. J. Am. Med. Dir. Assoc..

[23] Forough A.S., Wong S.Y.M., Lau E.T.L., Santos J.M.S., Kyle G.J., Steadman K.J., Cichero J.A.Y., Nissen L.M. (2018). Nurse experiences of medication administration to people with swallowing difficulties living in aged care facilities: A systematic review of qualitative evidence. JBI Database Syst. Rev. Implement. Rep..

[24] Loeb M.B., Becker M., Eady A., Walker-Dilks C. (2003). Interventions to prevent aspiration pneumonia in older adults: A systematic review. J. Am. Geriatr. Soc..

[25] Levac D., Colquhoun H., O’Brien K.K. (2010). Scoping studies: Advancing the methodology. Implement. Sci..

[26] Arksey H., O’Malley L. (2005). Scoping studies: Towards a methodological framework. Int. J. Soc. Res. Methodol. Theory Pr..

[27] Tricco A.C., Lillie E., Zarin W., O’Brien K.K., Colquhoun H., Levac D., Moher D., Peters M.D.J., Horsley T., Weeks L. (2018). PRISMA Extension for Scoping Reviews (PRISMA-ScR): Checklist and explanation. Ann. Intern. Med..

[28] Klor B.M., Milianti F.J. (1999). Rehabilitation of neurogenic dysphagia with percutaneous endoscopic gastrostomy. Dysphagia.

[29] Lin L.-C., Wang S.-C., Chen S.H., Wang T.-G., Chen M.-Y., Wu S.-C. (2003). Efficacy of swallowing training for residents following stroke. J. Adv. Nurs..

[30] Pelletier C.A., Lawless H.T. (2003). Effect of citric acid and citric acid-sucrose mixtures on swallowing in neurogenic oropharyngeal dysphagia. Dysphagia.

[31] Ebihara T., Ebihara S., Maruyama M., Kobayashi M., Itou A., Arai H., Sasaki H. (2006). A randomized trial of olfactory stimulation using black pepper oil in older people with swallowing dysfunction. J. Am. Geriatr. Soc..

[32] Taylor K.A., Barr S.I. (2006). Provision of small, frequent meals does not improve energy intake of elderly residents with dysphagia who live in an extended-care facility. J. Am. Diet. Assoc..

[33] Bautmans I., Demarteau J., Cruts B., Lemper J.-C., Mets T. (2008). Dysphagia in elderly nursing home residents with severe cognitive impairment can be attenuated by cervical spine mobilization. J. Rehabil. Med..

[34] Quagliarello V., Juthani-Mehta M., Ginter S., Towle V., Allore H., Tinetti M. (2009). Pilot testing of intervention protocols to prevent pneumonia in nursing home residents. J. Am. Geriatr. Soc..

[35] Hanson L.C., Carey T.S., Caprio A.J., Lee T.J., Ersek M., Garrett J., Jackman A., Gilliam R., Wessell K., Mitchell S.L. (2011). Improving decision-making for feeding options in advanced dementia: A randomized, controlled trial. J. Am. Geriatr. Soc..

[36] Park Y., Oh S., Chang H., Bang H.L. (2015). Effects of the evidence-based nursing care algorithm of dysphagia for nursing home residents. J. Gerontol. Nurs..

[37] Benigas J.E., Bourgeois M. (2016). Using spaced retrieval with external aids to improve use of compensatory strategies during eating for persons with dementia. Am. J. Speech-Lang. Pathol..

[38] Chen L.L., Li H., Lin R., Zheng J.H., Wei Y.P., Li J., Chen P., Chen H.Y. (2016). Effects of a feeding intervention in patients with Alzheimer’s disease and dysphagia. J. Clin. Nurs..

[39] Hollaar V.R.Y., van der Putten G.-J., Van der Maarel-Wierink C.D., Bronkhorst E.M., de Swart B.J.M., Creugers N.H.J. (2017). The effect of a daily application of a 0.05% chlorhexidine oral rinse solution on the incidence of aspiration pneumonia in nursing home residents: A multicenter study. BMC Geriatr..

[40] Jablonski R.A., Winstead V., Azuero A., Ptacek T., Jones-Townsend C., Byrd E., Geisinger M.L., Morrow C. (2017). Feasibility of providing safe mouth care and collecting oral and fecal microbiome samples from nursing home residents with dysphagia: Proof of concept study. J. Gerontol. Nurs..

[41] Zanini M., Bagnasco A., Catania G., Aleo G., Sartini M., Cristina M.L., Ripamonti S., Monacelli F., Odetti P., Sasso L. (2017). A Dedicated Nutritional Care Program (NUTRICARE) to reduce malnutrition in institutionalised dysphagic older people: A quasi-experimental study. J. Clin. Nurs..

[42] Hollaar V., van der Maarel-Wierink C., Van der Putten G.-J., de Swart B., de Baat C. (2015). Effect of daily application of a 0.05% chlorhexidine solution on the incidence of (aspiration) pneumonia in care home residents: Design of a multicentre cluster randomised controlled clinical trial. BMJ Open.

[43] Rudberg M.A., Egleston B.L., Grant M.D., Brody J.A. (2000). Effectiveness of feeding tubes in nursing home residents with swallowing disorders. J. Parenter. Enter. Nutr..

[44] Ballesteros-Pomar M.D., Cherubini A., Keller H., Lam P., Rolland Y., Simmons S.F. (2020). Texture-modified diet for improving the management of oropharyngeal dysphagia in nursing home residents: An expert review. J. Nutr. Health Aging.

[45] Cockburn N., Pateman K., Taing M.W., Pradhan A., Ford P.J. (2017). Managing the oral side-effects of medications used to treat multiple sclerosis. Aust. Dent. J..

[46] NICE (2014). Managing Medicines in Care Homes.

[47] Adhikari R., Tocher J., Smith P., Corcoran J., MacArthur J. (2014). A multi-disciplinary approach to medication safety and the implication for nursing education and practice. Nurse Educ. Today.

[48] Iyer A., Heathcote D. Safe Swallowing of Oral Liquid Medications in Patients with Dysphagia A Patient Quality & Safety Initiative—Trillium Health Partners. Proceedings of the CSHP 2015.

[49] Singh V., Brockbank M.J., Frost R.A., Tyler S. (1995). Multidisciplinary management of dysphagia: The first 100 cases. J. Laryngol. Otol..

[50] Duval M., Black M.A., Gesser R., Krug M., Ayotte D. (2009). Multidisciplinary evaluation and management of dysphagia: The role for otolaryngologists. J. Otolaryngol. Head Neck Surg.

[51] Aoki S., Hosomi N., Hirayama J., Nakamori M., Yoshikawa M., Nezu T., Kubo S., Nagano Y., Nagao A., Yamane N. (2016). The multidisciplinary swallowing team approach decreases pneumonia onset in acute stroke patients. PLoS ONE.

